# Animating and exploring phylogenies with fibre plots

**DOI:** 10.12688/f1000research.10274.3

**Published:** 2017-04-05

**Authors:** William D. Pearse

**Affiliations:** 1 Department of Biology & Ecology Center, Utah State University, Logan, UT, USA

**Keywords:** phylogeny, visualisation, 3D, fractal, animation, tree of life

## Abstract

Despite the progress that has been made in many other aspects of data visualisation, phylogenies are still represented in much the same way as they first were by Darwin. In this brief essay, I give a short review of what I consider to be some recent major advances, and outline a new kind of phylogenetic visualisation. This new graphic, the fibre plot, uses the metaphor of sections through a tree to describe change in a phylogeny. I suggest it is a useful tool in gaining an rapid overview of the timing and scale of diversification in large phylogenies.

A new generation of phylogeneticists are piecing together the entire tree of life, making vast phylogenies of millions of taxa ^1, 2^. Many have produced tree-like depictions of the relationships among species, both before [see 3] and after Darwin described the origin of species ^4^, but Haeckel’s drawings ^5^ are perhaps the most well-known. As our phylogenies become larger, a problem has emerged: humans cannot easily interpret phylogenies with millions of tips. In this brief essay, I will describe recent progress in the visualisation of phylogenies, and outline a new kind of plot—the “fibre plot”. My aim is not to write a review [ *c.f.* 6], but rather to provide an opinionated commentary on some major milestones in the progress of phylogenetic visualisation.

Haeckel’s phylogenies ^5^ are beautiful to look at, and convey the overall structure of a phylogeny well. Each minor branch rarely maps onto a particular species, but their presence reminds the reader of the ever-changing nature of diversification. Both Haeckel and Darwin convey two kinds of information in their visualisations: *time* through depth on the page, and *relatedness* through the branching structure itself. Haeckel is also notable for producing a series of phylogenies, each examining a finer phylogenetic scale. Haeckel grasped that humans cannot process the fine details of all species without becoming lost, and that a series of phylogenies provides the same information in a more digestible format than a single, large, fully-resolved tree.

The last one hundred years have seen transformative changes to phylogenetic inference [see 7], but the same is not true of phylogenetic visualisation. The pace of change of phylogenetic visualisation has not matched that of other aspects of statistical visualisation. A time-traveller from 1859 could decipher a phylogeny from 2017 with *On the Origin of Species* ^4^ as a guide, but the box-plots ^8^ and histograms ^9^ we rely on today would be foreign to them. Circular (“radial”) phylogenies are sometimes preferred when space is limited [ *e.g.*, 10, 11], and “magnifiers” in some computer programs highlight certain parts of the tree in more detail [ *e.g.*, 12], but for the most part any advances have been relatively minor.

A major innovation came when programs such as *Walrus* ^13, 14^ and *Paloverde* ^15^ allowed users to fly around phylogenies within 3D virtual spaces. Both are notable for presenting structure as something to be *explored*, not merely viewed, and that “ *a 3D world, offers visual cues that aid in navigation and display that is unavailable in strictly 2D versions of the same layout*” ^15^. The author of *Paloverde*, like Haeckel, recognised that scientists need to shift between finer and coarser phylogenetic scales when examining data, and so allowed users to collapse nodes at will. These programs were major advances in helping phylogeneticists conceptualise their own phylogenetic hypotheses.

At least as transformative was the release of *OneZoom* ^16^: a fractal phylogeny representation capable (theoretically) of displaying the entire tree of life on one page. *OneZoom* also requires the user to *explore* the tree, scanning up and down between finer and coarser details to make sense of the entire tree. Critically, *OneZoom*’s authors recognised that we are reaching the limits of what can be displayed in books: “ *[w]e now need to take the next step with a transition to data visualization that is optimized for interactive displays rather than printed paper*.” They suggest that the way to display the next generation of data is to use the next generation of technology.

A common thread running through these developments is their capacity to change the information displayed to the viewer, to better emphasise difference in structure across different phylogenetic depths. Consequently, I suggest the use of a new visualisation, the “fibre plot”, which is intended to leverage our natural ability to detect visual change through time. The fibre plot may be considered a horizontal slice through the tree of life, taken at whatever height (depth) the viewer requires ( Figure 1). By moving along the tree, from the root to the tip, viewers will see the relative width of each fibre, and so gauge the number of terminal tips subtending that clade. I emphasise that, while Figure 1 shows the underlying logic behind the plot, the “plot” should really be called an animation - it is most readily interpretable when the user watches a video composed of successive slices through the trunk of the tree. I suggest the animation, with frames recorded at equal intervals along that trunk, provides the viewer with an intuitive sense of the timing of the diversification of major clades. I have written R code to produce a fibre plot ( Supplementary File 1; to be released in the package pez ^17^), and an example of how it can be used to visualise the mammal tree of life ^18^ ( Supplementary File 2). The code can also be used with non-ulatrametric trees, where I find it particularly useful to represent the relative fraction of a tree that is extinct at any given time-point.

**Figure 1.**
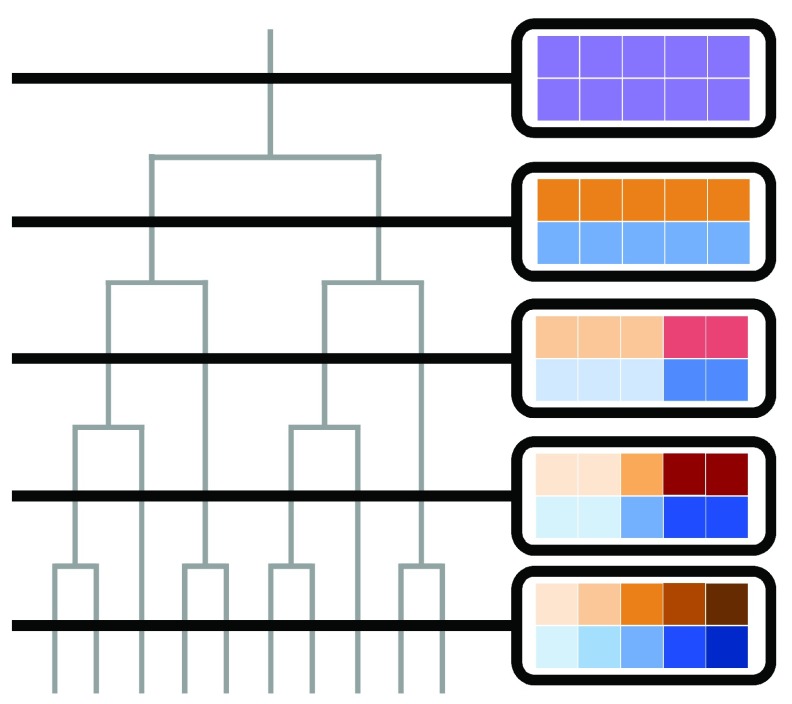
An explanation of a fibre plot. On the left, I show a phylogeny (in grey) with a series of slices cut through it (in black). To the right, I show views through those slices surrounded in black outlines: each of these slices forms the basis of a fibre plot. Within each slice, a square represents descendent tips, and colours of those squares represent the composition of clades within a particular time slice. Squares of the same colour form a “fibre” in the tree of life. A true fibre plot would be an animation of the transition between these slices, showing how the clades (fibres) that make up the tree split as diversification takes place. Alternate colouring schemes are possible for the fibres; the R implementation, by default, colours fibres according to clade age, and allows for different colouring schemes within a plot to highlight taxa of interest.

Despite humanity being closer than ever to a reliable tree of all life on Earth ^1, 2^, phylogenetic visualisation may seem like a niche topic. I strongly feel that phylogenetic visualisation is critical if we are to grasp the full extent of our planet’s biodiversity. Human activity has carelessly altered almost every aspect of our planet, and we must now live with the shame and hubris of a geologic age we named after ourselves ^19^. There has never been a greater need to find a way to show humanity our true place in the world. In whatever sense phylogeneticists have a duty, I believe it is ours to show the world that we are nothing more than a twig on a tree that we are cutting down.

## Supplementary files

**Supplementary File 1: R code for fibre plot**

.

**Supplementary File 2: Mammals.gif.** Animated fibre plot in Graphics Interchange Format (GIF) of a phylogeny of all extant mammals ^18^
