# Supplementary figures and images for: Animating and exploring phylogenies with fibre plots
Source: F1000Res. 2017 Apr 5;5:2790. Originally published 2016 Nov 29. [Version 3] doi: 10.12688/f1000research.10274.3 (PMC5389409; doi:10.12688/f1000research.10274.3)

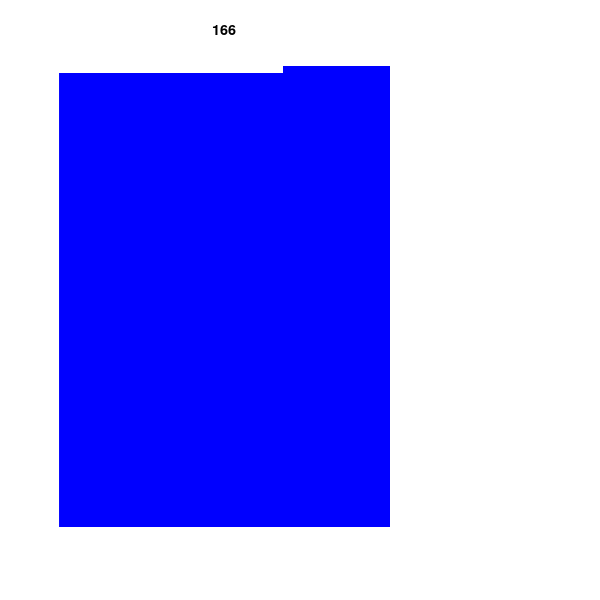

Supplement: Supplementary file 2 [file f1000research-5-12226-s0000.tgz › eec41406-2e5b-4b6e-9f4e-091319591806.gif]
