# Supplementary material for: Animating and exploring phylogenies with fibre plots
Source: F1000Res. 2017 Apr 5;5:2790. Originally published 2016 Nov 29. [Version 3] doi: 10.12688/f1000research.10274.3 (PMC5389409; doi:10.12688/f1000research.10274.3)
Supplement: Peer review history

# Peer review: Referee response for version 3

**DOI:** 10.5256/f1000research.12226.r21586

**Published:** 2017-04-10

**Authors:** Diego San Mauro, Ainhoa Agorreta

**Affiliations:** 1 Department of Zoology and Physical Anthropology, Complutense University of Madrid, Madrid, Spain

In our first review of this opinion article, we indicated several comments and suggestions for a few aspects that required further attention. We see that the author has incorporated most of the changes suggested (either by us or the other two referees) into the article, and we feel it looks now improved. In our opinion, this new version of the paper is satisfactory, and suitable for publication in F1000Research.

We have read this submission. We believe that we have an appropriate level of expertise to confirm that it is of an acceptable scientific standard.

# Peer review: Referee response for version 2

**DOI:** 10.5256/f1000research.11550.r19938

**Published:** 2017-02-17

**Authors:** Diego San Mauro, Ainhoa Agorreta

**Affiliations:** 1 Department of Zoology and Physical Anthropology, Complutense University of Madrid, Madrid, Spain

This paper presents a brief overview on phylogenetic visualization and introduces a novel approach for visualizing phylogenies (timetrees) using fibre plots. Given the rapid accumulation of phylogenetic information over the last years that has enabled the construction of massive trees (mega-phylogenies) containing millions of branches and leaves (taxa), the new visualization method appears to be interesting and with some potential. However, it is outlined only succinctly in the paper, and we feel that there are a few issues that require further attention. More discussion is needed on the specific applications and/on implementations of phylogenetic fibre plots compared to the other visualization approaches already available. For instance, what are the advantages of fiber plots over conventional phylogenetic plots in terms of comparing e.g. different topology sets? (as used for example in hypothesis testing). Also, what is the applicability (if any) of fiber plots for visualizing phylogenetic trees whose branches represent rate of evolution (e.g., substitutions/site) instead of time? (as in phylograms). Or, how do fibre plots deal with extinct branches? (as those displayed by extinct fossil lineages). Discussing these issues (among others) more in detail would make it easier for the reader to assess the breadth of novelty and usefulness of the new method for the general field of phylogenetics, and its applicability beyond the reconstruction of the timetree of life. As described in the current paper, it seems that fibre plots could be a complement, but not substitute of the other (more conventional) phylogenetic visualization approaches. The output of the fibre plot is colorful, but in general very difficult to interpret. In fact, interpreting the fibre plot output of very large phylogenies or even the tree of all life would be more difficult than interpreting more conventional approaches (those zooming in and out the phylogeny). Implementing some sort of labeling/cross-referencing with lists of taxa or even conventional phylogenetic trees live on the side could help in the precise interpretation of what is being displayed at each timeframe.

 There are also some additional issues that we want to mention:

- First paragraph: The sentence beginning "Many have..." needs some rewording... It is true that many have produced tree-like depictions of the relationships among species, but certainly not many before Darwin. So, please reword.
- Second paragraph: Please provide a reference for the sentence beginning "Haeckel grasped that humans...".
- Third paragraph: Besides Dendroscope, it would be fair to cite FigTree (http://tree.bio.ed.ac.uk/software/figtree/) as well in the last sentence.
- Fourth paragraph: It would be good to cite and discuss Walrus (http://www.caida.org/tools/visualization/walrus/) here as well. It appeared in 2001 (earlier than Paloverde) and allowed interactive 3D visualization of hierarchical graphs.
- Fifth paragraph: Please add references and expand the last statement about using Hilbert curves.
- Last paragraph: The last paragraph of the paper appears unnecessary and probably should be removed. Only the first sentence could be kept as part of the previous paragraph (as closing statement). If this sentence is retained, please keep in mind that phylogenies (e.g., the tree of life) are hypotheses. Therefore, it would be more appropriate to say "...being closer than ever to a reliable tree of all life", rather than "...being closer than ever to a true tree of all life".

We have read this submission. We believe that we have an appropriate level of expertise to confirm that it is of an acceptable scientific standard, however we have significant reservations, as outlined above.

# Response

**Published:** 2017-03-29

**Authors:** Will Pearse

**Affiliations:** Utah State University, USA

I thank you both for your comments, which have greatly improved the article. I'm particularly grateful that you mentioned Walrus; this was a huge oversight on my part, and I'm glad to have an opportunity to correct it! I apologise for that, due to space limitations of an opinion article (limited to 1000 words), I am not able to go into as much detail as I would like on some of the broader topics you raise. I have, however, significantly altered the code of the fibre plot following your suggestion about non-ultrametric phylogenies and highlighting particular taxa. In particular, your suggest of a phylogeny to the side of the plot, mirroring reviewer 2's suggestion, has greatly improved the figure. Thank you!

 Responding to each of your comments in turn:

- Branch lengths and extinct taxa. I have re-written the function so that it supports dated and undated trees, and highlights extinct taxa to show the time period within which they went extinct. I describe this in the penultimate paragraph of the manuscript.
- Ease of interpretation and suggestion of replacement of other phylogenies. I agree with the reviewers that this is not a replacement for a traditional visualisation; as I discuss in the text I find the visualisation captures well changes in timing and diversification more readily in extremely large phylogenies (e.g., the ~5000 taxon example I provide). I have followed the reviewers' suggestions and allowed the user to highlight clades and taxa of interest, which, along with the comments of reviewer 3, I hope make the plot easier to interpret.
- First paragraph: I respectfully disagree with your comment; the book by Pietsch I reference contains many examples of tree-like structures preceeding Darwin. I have altered the text to make my meaning clearer.
- Second paragraph: Thank you for this; I have added a reference earlier in the paragraph.
- Third paragraph: Thank you for this; I now cite FigTree and the R package ape.
- Fourth paragrph: Thank you for this; I now mention Walrus (citing a 1997 conference paper that describes what is essentially the same software under the name 'H3'), and cite another software package that converts phylogenies into Walrus format.
- Fifth paragraph: Thank you for this; having now experimented more thoroughly with the approach, I didn't find it aided interpretation. I have changed the code to alter the layout of the fibres, but I have dropped this reference from the text.
- Final paragraph: Thank you for this; I have made the changes your suggested.

# Peer review: Referee response for version 2

**DOI:** 10.5256/f1000research.11550.r19876

**Published:** 2017-02-13

**Authors:** Stephen A. Smith

**Affiliations:** 1 Department of Ecology and Evolutionary Biology, University of Michigan, Ann Arbor, MI, USA

Pearse presents a new means for visualizing large phylogenies called the fibre plot. The purpose of this plot is to better represent splits by different colors and shades. This is an interesting idea and is demonstrated with an accompanying animated gif. However, I am left wondering if there are significant insights gathered from this view of the mammal tree. The animation proceeds and areas of the graph change color. I understand why they change but don’t know where I am in the tree and what the significance of the change is in speed or area of the graph. The figure presented (Figure 1) shows a somewhat different view of the fibre plot as presented alongside the phylogeny. This makes me think that perhaps a more informative presentation would be the view of the phylogeny along with the fibre plot. Then the animation would follow a line that moves in a preorder fashion from the root to the tips. This would allow for a more direct comparison of the tree and the plot. Without this additional guide, I am not sure what to make of the animation. I don’t know where I am in the tree (in time or place) and I can’t “move around” in any particular way. I can also envision any number of statistics presented with the plot. 

 This is an interesting start of an idea but I think it needs a little more development before it would be useful for navigating the size of the tree intended by the author. However, there may be some interesting uses for this or something like it in the future. 

**Editorial comments**

 I recommend that the author edit the abstract. For example, the sentence “Despite the progress that has been made in the visualisation of information since Haeckel's time, phylogenetic visualisation has moved forward remarkably little.” seems to suggest that Haeckel was the first person to try and visualize data. While this may be accurate for some biological data, it is not true for data in general as cartographers have been trying to visualize information and data for centuries. The final sentence in the paragraph could also use some adjustments. While the statement is trying to convey a general sense of the importance of phylogenies, I am not certain that “our place” in the tree of life will dramatically change as a result of visualization of the data. I would also recommend changes to the intervening sentences.

 I would recommend some changes to the remaining text but won’t outline all of those here.

I have read this submission. I believe that I have an appropriate level of expertise to confirm that it is of an acceptable scientific standard, however I have significant reservations, as outlined above.

# Response

**Published:** 2017-03-29

**Authors:** Will Pearse

**Affiliations:** Utah State University, USA

Thank you for these suggestions. I have now altered the fibre plot code in exactly the way you suggested, adding a traditional phylogeny to the right-hand side of the animation that grows and matches colour with the fibre plot. I think it makes the plot much easier to interpret - thank you for this excellent suggestion! I have edited the abstract following your suggestions, and removed the final sentence from it entirely.

# Peer review: Referee response for version 2

**DOI:** 10.5256/f1000research.11550.r19939

**Published:** 2017-02-10

**Authors:** Rafael Zardoya

**Affiliations:** 1 Department of Biodiversity and Evolutionary Biology, Museo Nacional de Ciencias Naturales, Madrid, 28006, Spain

The paper entitled  “Animating and exploring phylogenies with fibre plots” by Pearse is an interesting contribution that proposes a new and distinct way to visualize phylogenetic trees. The new method propose by the author uses fibre plots to slice a phylogenetic tree from root to tips and visualize, as an animation, the cladogenetic process in time.

 As the author correctly argues, while it is now possible to reconstruct phylogenetic trees involving tens of thousands of species, visualization of such trees is complex and has not advanced at the same pace as probabilistic inference methodology. Hence, the challenge is set. There are many programs for visualizing trees but few have explored the need of dealing with large phylogenies. Different strategies have been proposed to represent phylogenies including the collapse of certain nodes, distortion of the view, and representation in 3D, but thus far, the most popular approach probably consists on zooming in and out the phylogeny (OneZoom, Rosindell *et al.* 2012) using appropriate tools (e.g., a tablet). These viewers are complemented with others that allow incorporating other information pertinent to the phylogeny (e.g., iTOL, Letunic and Bork 2016).

 The proposal here presented explores in a very different direction. While the idea of looking at different temporal slices in the phylogeny to get a feeling of the timing of diversification of the different clades is original, I think it is too preliminary in the present contribution. The video composed of successive slices shows in different colors how a single (ancestral) lineage is successively split into many but the viewer is unable to discern to which exact descendant lineages is looking at, as there are no labels. Moreover, at some point the number of splits (and colors) is too large to obtain useful information from the animation. As presently devised, the analysis of different clades will render very similar plots, which will be difficult to interpret (beyond seen an increase in the number of lineages) and compare. If the author wants this tool to be widely used, he should make the final outcome more appealing and understandable (e.g., perhaps a grid plot with labels of each lineage in the corresponding axis would help following which lineages and their ancestors are diverging) by peers from other fields than phylogenetics and by the general public.

 Minor changes:

- The author mentions that PaloVerde in 2006 was the first 3D phylogeny viewer to his knowledge. I think he should check the Walrus graph visualisation tool by Hughes *et al.* 2004
- Close brackets after [see 6]

I have read this submission. I believe that I have an appropriate level of expertise to confirm that it is of an acceptable scientific standard, however I have significant reservations, as outlined above.

## References

[1] RosindellJHarmonLJ: OneZoom: a fractal explorer for the tree of life. *PLoS Biol*.2012;10(10) : 10.1371/journal.pbio.1001406 e1001406 10.1371/journal.pbio.1001406 23091419PMC3472976

[2] LetunicIBorkP: Interactive tree of life (iTOL) v3: an online tool for the display and annotation of phylogenetic and other trees. *Nucleic Acids Res*.2016;44(W1) : 10.1093/nar/gkw290 W242-5 10.1093/nar/gkw290 27095192PMC4987883

[3] HughesTHyunYLiberlesDA: Visualising very large phylogenetic trees in three dimensional hyperbolic space. *BMC Bioinformatics*.2004;5: 10.1186/1471-2105-5-48 48 10.1186/1471-2105-5-48 15117420PMC419335

# Response

**Published:** 2017-03-29

**Authors:** Will Pearse

**Affiliations:** Utah State University, USA

Thank you for these comments. I hope the changes I have made address some of your concerns about interpretation, all of which I think are legitimate. I have added the capacity to label (and track) particular species and clades through the animation, and have added an optional display of the phylogeny to the side of the animation. I hope the reviewer agrees that this addresses their concerns. Your comments about the colouring scheme, in particular, were very useful - I now colour everything according to clade age, which I hope you will agree makes for a much more informative plot. Thank you also for mentioning Walrus - omitting this was a huge mistake, and I'm grateful you've corrected me.
